# A Case of Brugada Pattern Associated with Adrenal Insufficiency

**DOI:** 10.7759/cureus.2752

**Published:** 2018-06-06

**Authors:** Corina Iorgoveanu, Ahmed Zaghloul, Aakash Desai, Kathir Balakumaran, Muhammad Y Adeel

**Affiliations:** 1 Internal Medicine, University of Connecticut Health Center, Farmington, USA; 2 Cardiology, University of Connecticut Health Center, Farmington, USA; 3 Internal Medicine, Yale New Haven Health at Bridgeport Hospital, Bridgeport, USA

**Keywords:** brugada syndrome, adrenal insufficiency

## Abstract

Brugada syndrome (BrS) is an inherited channelopathy disease, caused by genetic changes in transmembrane ion channels. It has an increased risk of sudden cardiac death (SCD) in the absence of a structural heart disease. We report a case in which the presenting electrocardiogram (EKG) exhibited a type 1 Brugada-like pattern during an adrenal crisis with transformation into a type 2 Brugada-like pattern as the crisis improved.

## Introduction

Brugada syndrome (BrS) is an inherited disease characterized by a coved-type ST-segment elevation in the right precordial leads (V1–V3) and an increased risk of sudden cardiac death (SCD) in the absence of a structural heart disease [1]. Common triggers for the Brugada pattern include exercise, fever, ischemia, and certain medications. Here, we report a case of an under-estimated precipitant for Brugada pattern: adrenal crisis.

## Case presentation

**Figure 1:**
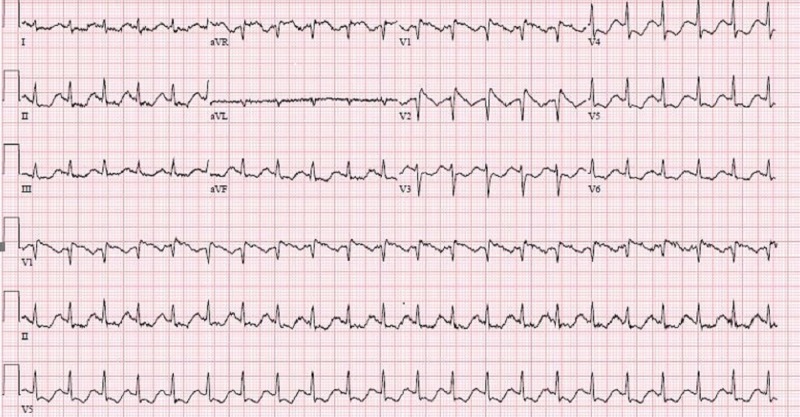
ECG on arrival: normal sinus rhythm at a heart rate of 125 bpm, coving pattern in V1 and V2 suggestive of Brugada type 1 pattern, subtle ST depressions in antero-lateral leads

A 32-year-old female with no previous medical history presented to the emergency department (ED) with weakness and fever, along with diarrhea and vomiting for one day. She had no complaints of chest pain, shortness of breath, chills, headaches, dizziness, or palpitations on arrival. Family history, social history, and past surgical history were all unremarkable. However, she had a history of sudden cardiac death in her family. On arrival in the ED, she had a temperature of 103.1 Fahrenheit, tachycardia at 131 beats per minute (bpm), with a blood pressure of 65/38 mmHg. On physical examination, she was oriented only to self and disoriented to time, place, and person; the physical examination did not show any other significant findings. Laboratory data demonstrated an acute kidney injury with a creatinine of 1.7 mg/dl. Cardiac enzymes were negative. The initial electrocardiogram (ECG) was notable for right bundle branch block (RBBB) with coved Brugada-type ST-T wave changes in V1 and V2 along with diffuse ST depressions (Figure 1).

The patient required admission to the intensive care unit (ICU) given her hemodynamic instability and the need for pressor support with norepinephrine. Further investigations revealed a low serum cortisol level (2.1 UG/DL) and a low adrenal corticotropic hormone (ACTH) (<5pg/ml). A cosyntropin stimulation test was performed next, which was consistent with secondary adrenal insufficiency (AI). Steroid supplementation was initiated with a significant improvement in her clinical picture.

**Figure 2:**
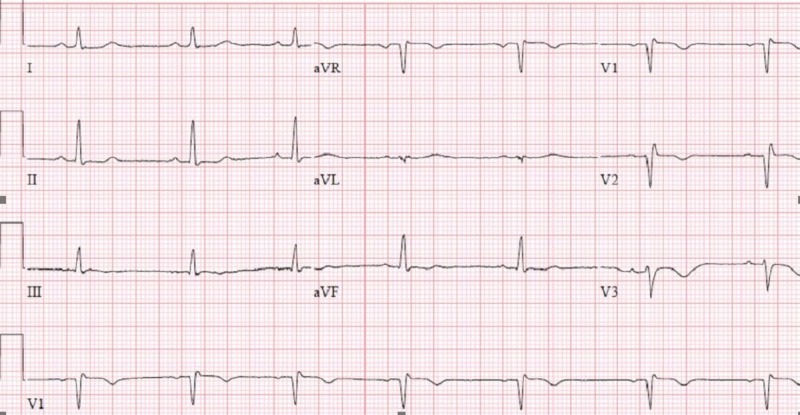
Repeat ECG: normal sinus rhythm at a rate of 62 bpm with incomplete RBBB and mild coving consistent with a type 2 pattern RBBB: right bundle branch block

Repeat ECG revealed the resolution of the diffuse ST depressions and an incomplete RBBB with mild coving consistent with a type II pattern (Figure 2).

No arrhythmias were recorded throughout the admission. A transthoracic echocardiography (TTE) was conducted, which yielded normal results. At discharge, the patient was advised to have an immediate intervention with antipyretics for any future febrile episodes and to avoid certain medications. She was discharged on steroid supplementation and with close follow-up with cardiology.

## Discussion

Understanding the conditions that can disclose the hidden Brugada syndrome is critical, especially for the patients, so that they may be informed to promptly request medical attention. The diagnosis of BrS is often missed due to its dynamic nature. It has been suggested that the number of asymptomatic Brugada patients diagnosed currently are only the tip of the iceberg since many more would have been discovered if their ECGs were recorded during febrile illnesses.

A variety of triggers were proven to unmask the Brugada-like pattern (BrP) (fever, electrolyte imbalance, vagal stimulation) and trigger ventricular arrhythmias [2]. Hyperkalemia, a common electrolyte disturbance in AI, produces BrP by decreasing the resting membrane potential, which leads to the domination of the outward potassium current. This is more active in the epicardial cells of the right ventricle [3]. AI can also be associated with fever, which acts as a trigger, hence, uncovering the underlying BrS. Adler et al. found that in ECG recordings from 402 febrile patients, eight (2%) had a type 1 BrP [4]. All patients with fever-induced type 1 BrP were asymptomatic and remained so during follow-up. However, the consensus is that fever-induced BrS is a malignant disease. Junttila et al. described 16 patients with fever-induced type 1 BrP, where 10 had a cardiac arrest or syncope [5]. Fever was the precipitating factor of arrhythmias in 18% of Brugada patients presenting with cardiac arrest [6].

Current practical guidelines and consensus recommend the implantation of an implantable cardioverter defibrillator (ICD) in patients surviving a sudden cardiac death (SCD) (class I) and those with syncope and spontaneous type 1 ECG (class IIa). Clinical guidelines lack specific recommendations for asymptomatic patients. Spontaneous type 1 ECG, inducible ventricular arrhythmias during an electrophysiological study, and the presence of sinus node dysfunction can identify patients at a higher risk. Novel risk markers might also help in their management [1].

## Conclusions

To our knowledge, very few case reports exist regarding adrenal crisis as a possible trigger of the Brugada syndrome. Adrenal insufficiency should be considered a differential diagnosis in patients with these ECG findings in appropriate clinical scenarios.
